# Ecological traits predict extinction risk of the world’s traded carnivores

**DOI:** 10.1371/journal.pone.0362196

**Published:** 2026-11-05

**Authors:** Erik Joaquín Torres-Romero, Maximilian L. Allen, Vincent Nijman, Moreno Di Marco, Christopher J. O’Bryan, Vincenzo Penteriani

**Affiliations:** 1 Ingeniería en Biotecnología-Universidad Politécnica de Puebla, San Mateo Cuanalá, Juan C. Bonilla, C.P 72640, Puebla, Mexico; 2 Tecnológico Nacional de México campus Zacapoaxtla. Subdirección de Investigación y Posgrado, División de Biología, Carretera Acuaco-Zacapoaxtla Km. 8, Col. Totoltepec, Zacapoaxtla, C.P. 73680, Puebla, Mexico; 3 Red Nacional en Tecnologías Ambientales y Biotecnológicas para los Ecosistemas, Tecnológico Nacional de México, Puebla, Mexico; 4 Illinois Natural History Survey, Prairie Research Institute, University of Illinois, Champaign, Illinois, United States of America; 5 School of Biological and Medical Sciences, Oxford Brookes University, Oxford, United Kingdom; 6 IUCN/SSC Bear Specialist Group, Gland, Switzerland; 7 EcoVerde Global Consulting, Sacramento, California, United States of America; 8 Department of Biology and Biotechnologies ‘Charles Darwin’, Sapienza University of Rome, Rome, Lazio, Italy; 9 System Earth Science, Maastricht University, Venlo, the Netherlands; 10 Department of Evolutionary Ecology, National Museum of Natural Sciences (MNCN), Spanish National Research Council (CSIC), Madrid, Spain; University of the Faroe Islands: Frodskaparsetur Foroya, FAROE ISLANDS

## Abstract

Wildlife trade is a major driver of biodiversity loss, yet how trade interacts with species’ ecological traits and extinction risk remains underexplored. Carnivores warrant special attention because they are disproportionately exploited and vulnerable to extinction compared to other taxa. We analyzed 171,637 international trade records spanning 50 years (1975–2024) for 129 carnivore species listed under the Convention on International Trade in Endangered Species of Wild Fauna and Flora (CITES). We analyzed trade volumes across the globe and then used logistic regression to evaluate associations between ecological traits and extinction risk, as well as family-level differences in extinction risk. Trade volumes were dominated by exports from North America and Europe and imports concentrated in East Asia, highlighting persistent transcontinental trade. Logistic regression identified longer generation length and greater dispersal distance as positively associated with higher extinction risk, whereas broader habitat breadth was associated with lower extinction risk. Among carnivore families, only Ursidae (bears) exhibited significantly greater odds of being threatened relative to the Canidae (canids) reference family. Our findings demonstrate that ecological traits are associated with extinction risk within CITES-listed carnivores, supporting the use of trait-based approaches as a complementary tool for conservation assessment and monitoring of internationally traded species.

## Introduction

Understanding why species become vulnerable to extinction is a central challenge of 21st-century conservation science [1], as species extinctions accelerate globally under escalating anthropogenic pressures [2]. Among terrestrial vertebrates, mammals have faced a disproportionate burden of human expansion [3,4], with carnivores emerging as a critical sentinel group due to widespread declines, which lead to a profound loss of ecosystem functions and services [5,6]. The inherent vulnerability of carnivores arises from a convergence of intrinsic ecological traits and external pressures: they typically require extensive home ranges, exhibit slow life histories, and frequently experience direct persecution due to real or perceived conflicts with humans and livestock [7,8]. These same characteristics also underpin their ecological importance as keystone regulators of prey populations and trophic dynamics, such that their decline can precipitate cascading effects leading to ecosystem degradation [9,10]. This combination of high ecological importance and elevated extinction risk underscores the urgent need for proactive, mechanism-based conservation strategies [5,11].

Global wildlife trade has become one of the principal anthropogenic pressures accelerating biodiversity loss, affecting over one-third of threatened terrestrial vertebrates and showing little evidence of long-term sustainability [12,13]. While attention has traditionally focused on iconic megafauna or species harvested for food and traditional medicine, a growing body of evidence reveals that a broad spectrum of carnivore species is increasingly exploited—both legally and illegally—for luxury products, medicinal use, and the pet trade [14–17, Fig 1]. Wildlife trade rarely operates in isolation; instead, it commonly interacts with habitat loss, fragmentation, and other anthropogenic pressures jointly driving populations beyond demographic recovery thresholds [18,19].

**Fig 1:**
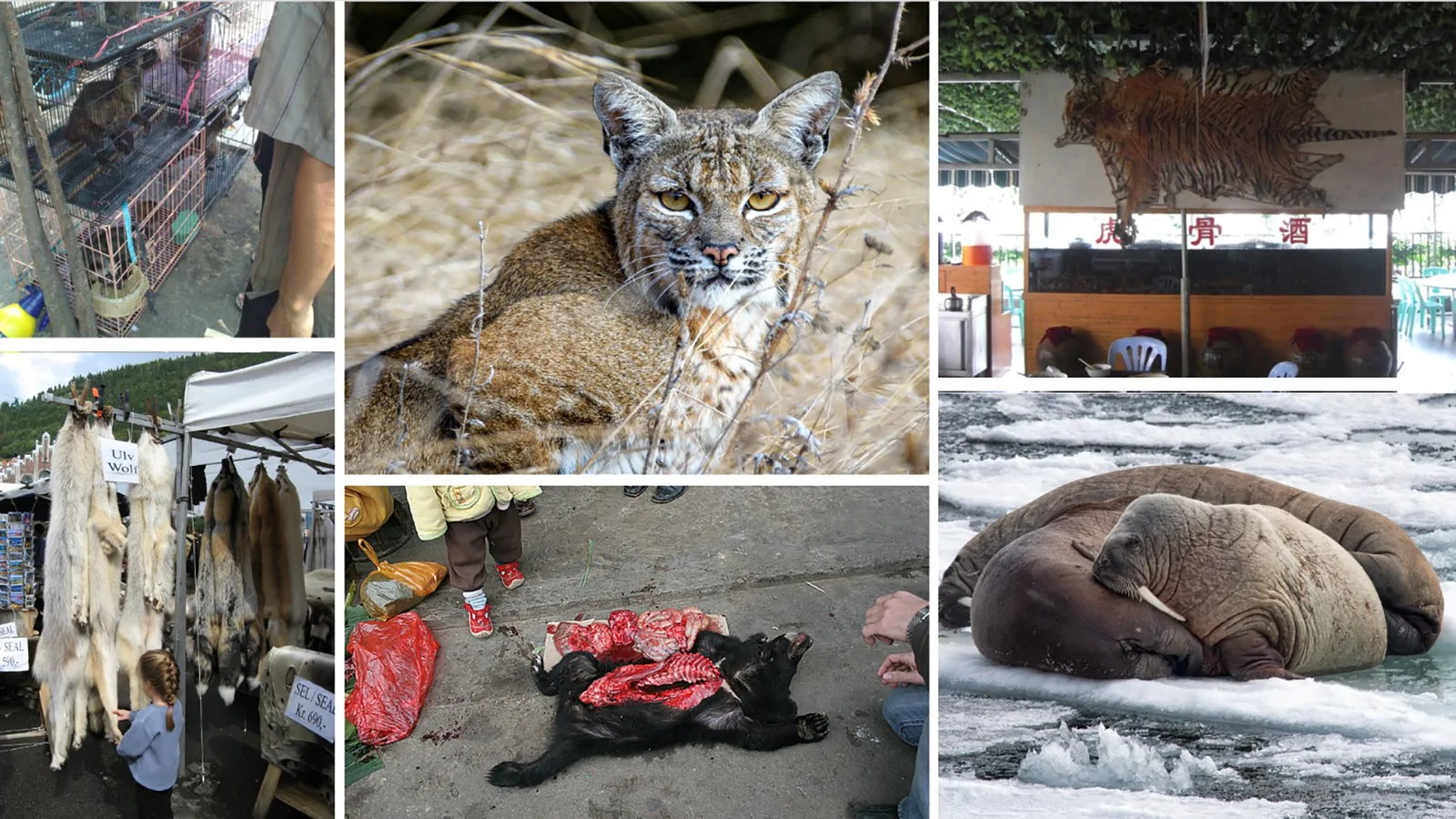
Representative examples of carnivore species involved in global wildlife trade. Trade in carnivores, from top left, clockwise: Common palm civet *Paradoxurus hermaphroditus* [Least Concern], Indonesia, traded as exotic pets and for the production of civet coffee (Vincent Nijman); bobcat *Lynx rufus* [Least Concern], USA, traded for their skins (Becky Matsubara, CC-BY 2.0); tiger *Panthera tigris* [Endangered], Myanmar, traded for medicine, skins and meat (Vincent Nijman); walrus *Odobenus rosmarus* [Vulnerable], Russia, traded for its tusks (Christopher Michel, CC-BY 2.0); Asiatic black bear *Ursus thibetanus* [Vulnerable], Myanmar, traded for meat and medicine (Vincent Nijman); wolf *Canis lupus* [Least Concern], red fox *Vulpes vulpes* [Least Concern] and unidentified seal, Norway, traded for their skins (Natalie Maynor, CC-BY 2.0).

Despite international regulatory mechanisms such as the Convention on International Trade in Endangered Species of Wild Fauna and Flora (CITES), enforcement remains uneven and listing decisions often lag behind rapidly emerging trade threats [20]. However, while ecological traits strongly mediate species’ sensitivity to anthropogenic exploitation and environmental change [21], international governance frameworks rarely incorporate biological vulnerability into regulatory prioritization [22,23]. Whether ecological traits consistently predict extinction vulnerability among CITES-listed carnivores remains poorly understood. Addressing this gap could complement existing conservation assessments and improve evidence-based trade regulation.

Trait-based modelling has become an important framework for predicting extinction risk, particularly where detailed demographic data are lacking across taxa and geographic regions [8,24,25]. Life-history traits—including body size, reproductive output, dispersal capacity, and population density—have consistently been associated with extinction vulnerability across vertebrates [24,26]. Although large-bodied carnivores with slow reproductive rates, extensive spatial requirements, and low population densities tend to be disproportionately threatened [3,5,27,28], the order Carnivora also includes numerous smaller-bodied lineages (e.g., weasels [Mustelidae], civets [Viverridae], mongooses [Herpestidae], and Malagasy carnivorans [Eupleridae]) that experience substantial exploitation through the international wildlife trade despite exhibiting distinct ecological strategies [29]. However, most extinction risk models have focused on broad-scale predictors without explicitly evaluating their intersection with international trade regulations [30,31]. Given the continued growth and global scale of wildlife trade, identifying trait-mediated pathways of vulnerability has become an increasingly important conservation priority [32–34].

The order Carnivora encompasses remarkable ecological diversity, including families that differ markedly in their ecological roles, life-history strategies, geographic distributions, and evolutionary histories [35,36]. From large-bodied, wide-ranging canids and felids to smaller-bodied mustelids, viverrids, herpestids, and other lineages, ecological diversity across Carnivora likely shapes family-specific exposure to wildlife trade and other anthropogenic threats [37,38]. For instance, while large cats and bears are frequently traded for skins and traditional medicine, smaller carnivores may be sold as exotic pets, for medicinal use or wild meat [39–42]. These distinctions underscore the need to analyze extinction risk both at the species level and across taxonomic groupings to detect clade-level vulnerabilities and prioritize conservation efforts accordingly [43].

A persistent obstacle to conservation planning is the scarcity of demographic information for many traded carnivore species, particularly small-bodied or poorly monitored taxa that remain underrepresented in monitoring programs despite potentially high trade pressure in tropical regions [44,45]. In such contexts, trait-based predictive models can serve as early warning systems, identifying species that possess characteristics linked to high extinction risk prior to formal threat assessments [46]. Trait-based models therefore provide a practical means of identifying species likely to be vulnerable before population declines become irreversible or formal threat assessments are updated [47,48]. Focusing on ecological traits of CITES-listed carnivores offers a complementary framework for improving listing decisions, prioritizing monitoring efforts, and guiding proactive conservation actions.

In this study, we integrated species-level life-history traits, IUCN Red List assessments, and five decades of CITES trade records to evaluate patterns of extinction vulnerability among globally traded carnivores. Specifically, we asked three questions: (1) which ecological and life-history traits are associated with higher extinction risk in traded species? (2) Do ecological and life-history traits vary across IUCN extinction-risk categories and population trend groups? And (3) does the probability of being threatened differ among traded carnivore families? By integrating trait-based modelling with global wildlife trade data, our study provides an evidence-based framework for evaluating ecological correlates of extinction vulnerability and informing conservation prioritization under international wildlife trade regulation.

## Materials and methods

### Species-level ecological and trade data compilation

We compiled a comprehensive global dataset comprising 129 CITES-listed carnivore species (Order: Carnivora) by integrating international trade records, life-history traits, and conservation status from three authoritative databases. The IUCN Red List was used to obtain conservation assessments, the CITES Trade Database provided international trade records, and the COMBINE database [49] supplied species life-history traits. First, we extracted species-specific threat assessments and taxonomic validations from the IUCN Red List (www.iucnredlist.org, accessed July 2025). The IUCN Red List was selected because the objective of this study was to identify life-history correlates of extinction vulnerability. Although all analyzed species are CITES-listed, CITES appendices are trade-regulatory classifications rather than measures of extinction risk and therefore were not appropriate as the response variable for our analyses. We extracted IUCN threat categories—Critically Endangered (CR), Endangered (EN), Vulnerable (VU), Near Threatened (NT), and Least Concern (LC)—together with population trajectory trends (Increasing, Stable, Decreasing, Unknown). We excluded Data Deficient (DD) species because insufficient information precludes reliable assessment of their extinction risk, making them unsuitable for the binary classification framework used in our analyses. We then compiled international trade records from the CITES Trade Database (trade.cites.org), which provides the definitive documentation of legally reported international wildlife commerce regulated under the Convention. We processed all available records spanning 1975–2024 (data for 2025 were pending at the time of analysis), including live individuals, skins, trophies, and body parts. We acknowledge that CITES coverage is restricted to international transactions and excludes domestic exchanges; however, with 185 countries party to CITES as of 2025, the database provides the most standardized and globally comparable measure of international wildlife trade currently available. Third, we integrated life-history and demographic trait data from the COMBINE database [49], a curated source of validated mammalian biological attributes. The analyzed ecological and life-history traits included adult body mass, generation length, maximum longevity, litter size, litters per year, dispersal distance, population density, and habitat breadth.

To ensure a consistent analytical framework, the final dataset was restricted to 129 CITES-listed carnivore species (representing 44% of the 291 extant species) distributed across 15 families: Ailuridae, Canidae, Eupleridae, Felidae, Herpestidae, Hyaenidae, Mephitidae, Mustelidae, Odobenidae, Otariidae, Phocidae, Prionodontidae, Procyonidae, Ursidae, and Viverridae. Restricting the dataset to CITES-listed species ensured that all analyses were conducted within a common international trade-regulatory context while retaining broad taxonomic and ecological representation. This framework provided the basis for evaluating associations between selected life-history traits and extinction vulnerability among CITES-listed carnivore species.

### Trait imputation and data preprocessing

We addressed missing values in continuous ecological traits using single median imputation. For each trait, missing observations were assigned the overall median calculated across the dataset, rather than medians calculated separately by genus or family. This approach retained species with incomplete trait data but did not account for uncertainty associated with imputed values [50,51]. Categorical variables, including IUCN Red List status and population trend, were not imputed because they represent observed classification data rather than continuous biological traits. Species with missing values in categorical variables were excluded from analyses requiring those variables; therefore, no categorical variables were imputed. Since all analyzed species were already restricted to CITES-listed carnivores, CITES inclusion was constant across the dataset and therefore was not considered in the imputation procedure. We subsequently standardized all continuous traits using z-transformations (mean = 0, SD = 1) to facilitate coefficient comparability and enhance model convergence. We performed data wrangling, imputation, and transformation steps in R (version 4.5.1; [52]) using the packages *dplyr* [53], *tidyr* [54], and *Hmisc* [55].

### Trait variation across threat categories

To evaluate whether ecological traits varied across IUCN threat categories and population trends, we used Kruskal-Wallis rank-sum tests because several variables exhibited non-normal distributions and heterogeneous variances. Separate tests were performed for each ecological trait across IUCN categories, and trait distributions were visualized using boxplots. All analyses were conducted in R using the *FSA* [56] and *ggpubr* [57] packages.

### Logistic regression and multicollinearity control

To identify the ecological determinants most strongly associated with extinction risk, we implemented binomial logistic regression models using IUCN Red List status as a binary dependent variable: threatened species (CR, EN, VU) were coded as 1, while non-threatened species (NT, LC) were coded as 0. We fitted an initial binomial logistic regression model using the *glm* function with a logit link. Multicollinearity among predictors was assessed using Variance Inflation Factors (VIFs), adopting a conservative exclusion threshold of VIF > 5 [58,59]. Candidate predictors were subjected to bidirectional stepwise model selection based on Akaike's Information Criterion (AIC), and the subset of predictors retained after model selection was used to construct the final logistic regression model. This procedure ensured both numerical stability and ecological interpretability. To evaluate predictive performance and robustness, we computed accuracy, sensitivity, specificity, Cohen’s kappa, and the Area Under the Receiver Operating Characteristic Curve (AUC), utilizing the *caret* [60], *pROC* [61], and *yardstick* [62] packages. We interpreted Odds Ratios (ORs) with 95% confidence intervals to quantify the magnitude and directionality of trait effects; where OR > 1 indicated elevated extinction risk, while OR < 1 suggested a protective effect.

### Family-level differences in extinction risk

To evaluate whether the probability of being threatened differed among carnivore families, we fitted a bias-reduced binomial logistic regression using taxonomic family as the predictor and IUCN threat status as the binary response variable (threatened = CR, EN, VU; non-threatened = NT, LC). Because some carnivore families were represented by relatively few species, we used bias-reduced maximum likelihood estimation implemented in the *brglm2* package [63], which implements the bias-reduction methodology of [64], to reduce small-sample bias and separation issues. Canidae was specified as the reference category for the family factor. Regression coefficients, odds ratios (ORs), and 95% confidence intervals were used to quantify differences in extinction risk among carnivore families relative to the reference category.

## Results

### Global trade dynamics of CITES-listed carnivores

Across 171,637 international trade records for 129 CITES-listed carnivore species spanning 1975–2024, trade volumes were unevenly distributed across countries, with export activity concentrated primarily in North America, Europe, and parts of Asia (Fig 2a). The United States, Canada, South Africa, Russia, Germany, the United Kingdom, and France accounted for the highest reported export activity. Import activity showed a broadly similar geographic pattern, with the United States, China, Japan, Canada, and several European countries representing the principal importing nations (Fig 2b). Overall, the global trade network of CITES-listed carnivores was dominated by North American, European, and Asian countries.

**Fig 2:**
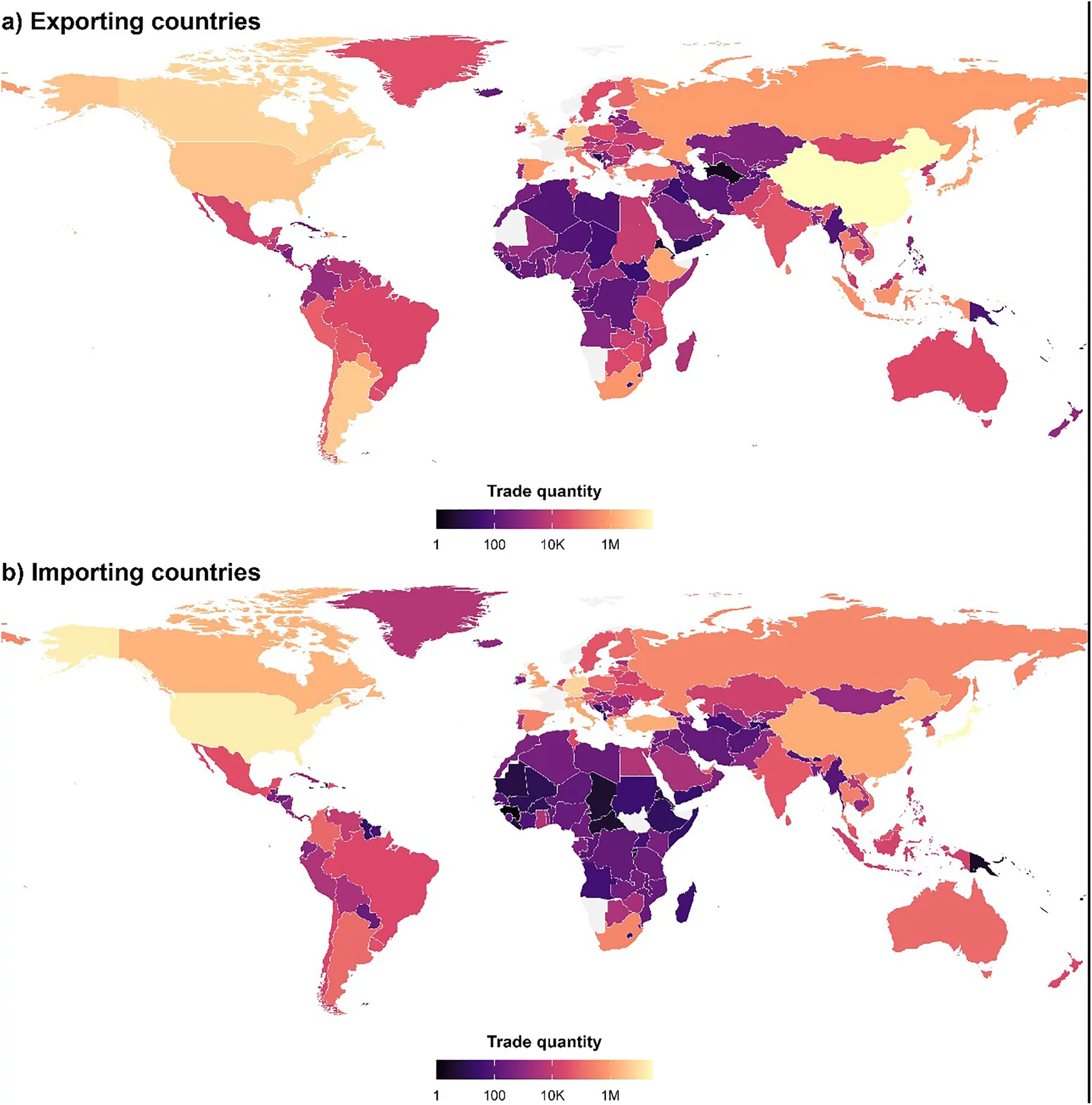
Global distribution of international trade in CITES-listed carnivores (1975–2024). Countries are colored according to the cumulative reported trade quantity for (a) exports and (b) imports, ranging from dark purple (low activity) to bright yellow (greater activity). The figure was created by the authors in R using the packages *ggplot2*, *sf*, and *rnaturalearth*, based on processed CITES trade records. The underlying basemap is derived from the Natural Earth public-domain dataset (CC0), distributed through the *rnaturalearth* package.

### Functional trait patterns across extinction risk and population trends

Ecological and life-history traits showed variable patterns across IUCN extinction-risk categories and population trend groups. Kruskal-Wallis tests identified significant differences among IUCN categories for adult body mass (p = 0.025), litter size (p = 0.034), and dispersal distance (p = 0.0018), whereas generation length, maximum longevity, litters per year, and population density did not differ significantly (Table 1). Although substantial overlap was evident among IUCN categories, species differing in extinction risk also differed in body mass, litter size, and dispersal distance (Fig 3). Likewise, species with different population trends exhibited contrasting distributions for several ecological traits, particularly body mass, litter size, and dispersal distance (Fig 4). Overall, only a subset of ecological and life-history traits varied significantly across IUCN extinction-risk categories, whereas the remaining traits showed broadly similar distributions despite visible variation among groups.

**Table 1:** Results of Kruskal-Wallis tests evaluating differences in ecological and life-history traits among IUCN extinction-risk categories for CITES-listed carnivores. Significant p-values (<0.05) are shown in bold.

Trait	χ²	df	p-value
Adult body mass (g)	11.13	4	**0.025**
Generation length (days)	7.22	4	0.125
Maximum longevity (days)	9.06	4	0.060
Litter size	10.41	4	**0.034**
Litters per year	4.09	4	0.394
Dispersal distance (km)	17.18	4	**0.0018**
Population density (ind/km²)	4.72	4	0.317

**Fig 3:**
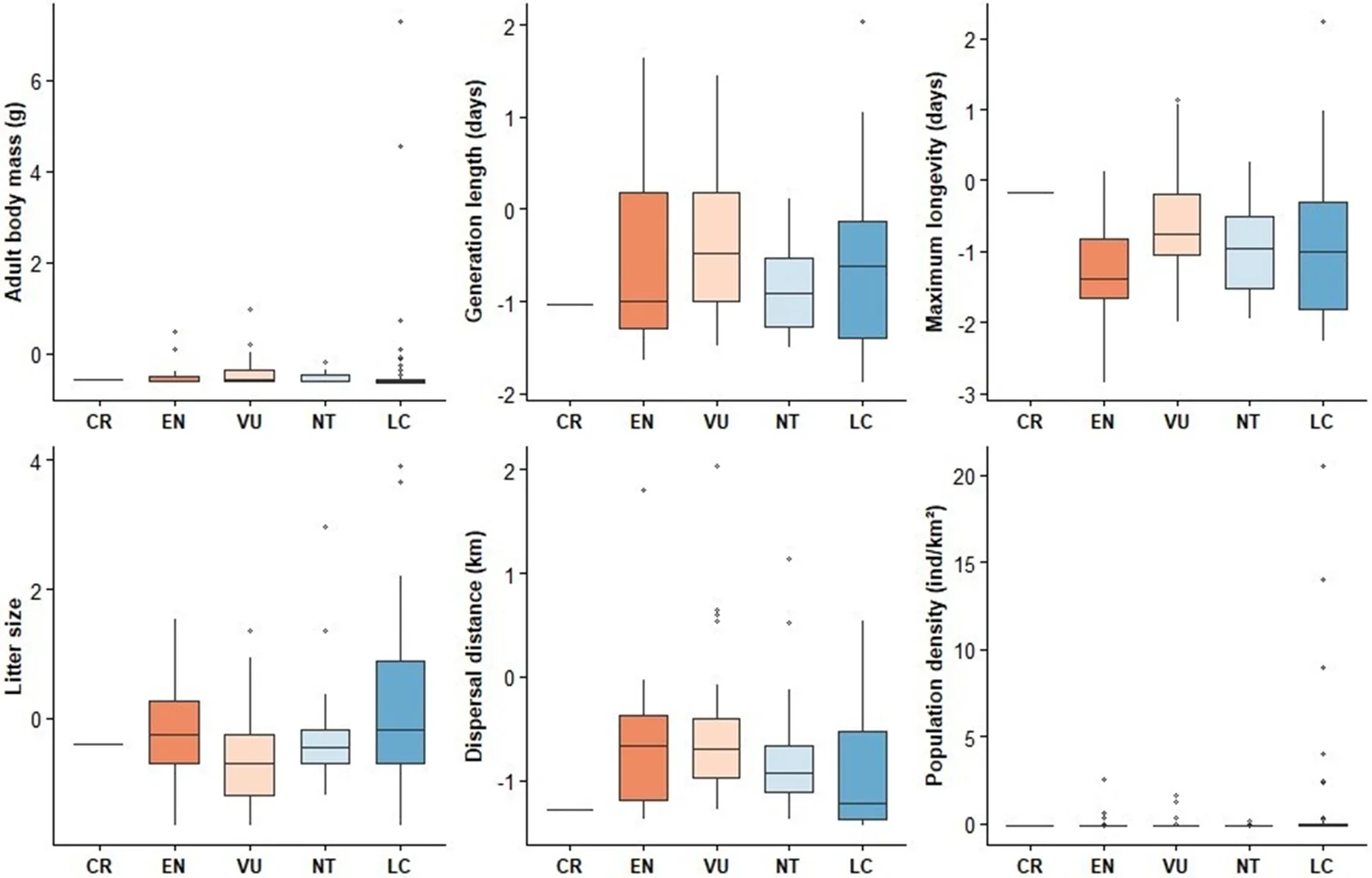
Variation in ecological and life-history traits across IUCN extinction-risk categories. Boxplots illustrating variation in adult body mass, generation length, maximum longevity, litter size, dispersal distance, and population density among CITES-listed carnivores classified as Critically Endangered (CR), Endangered (EN), Vulnerable (VU), Near Threatened (NT), and Least Concern (LC). Boxes represent the interquartile range (IQR), horizontal lines indicate medians, whiskers extend to 1.5 × IQR, and points represent outliers.

**Fig 4:**
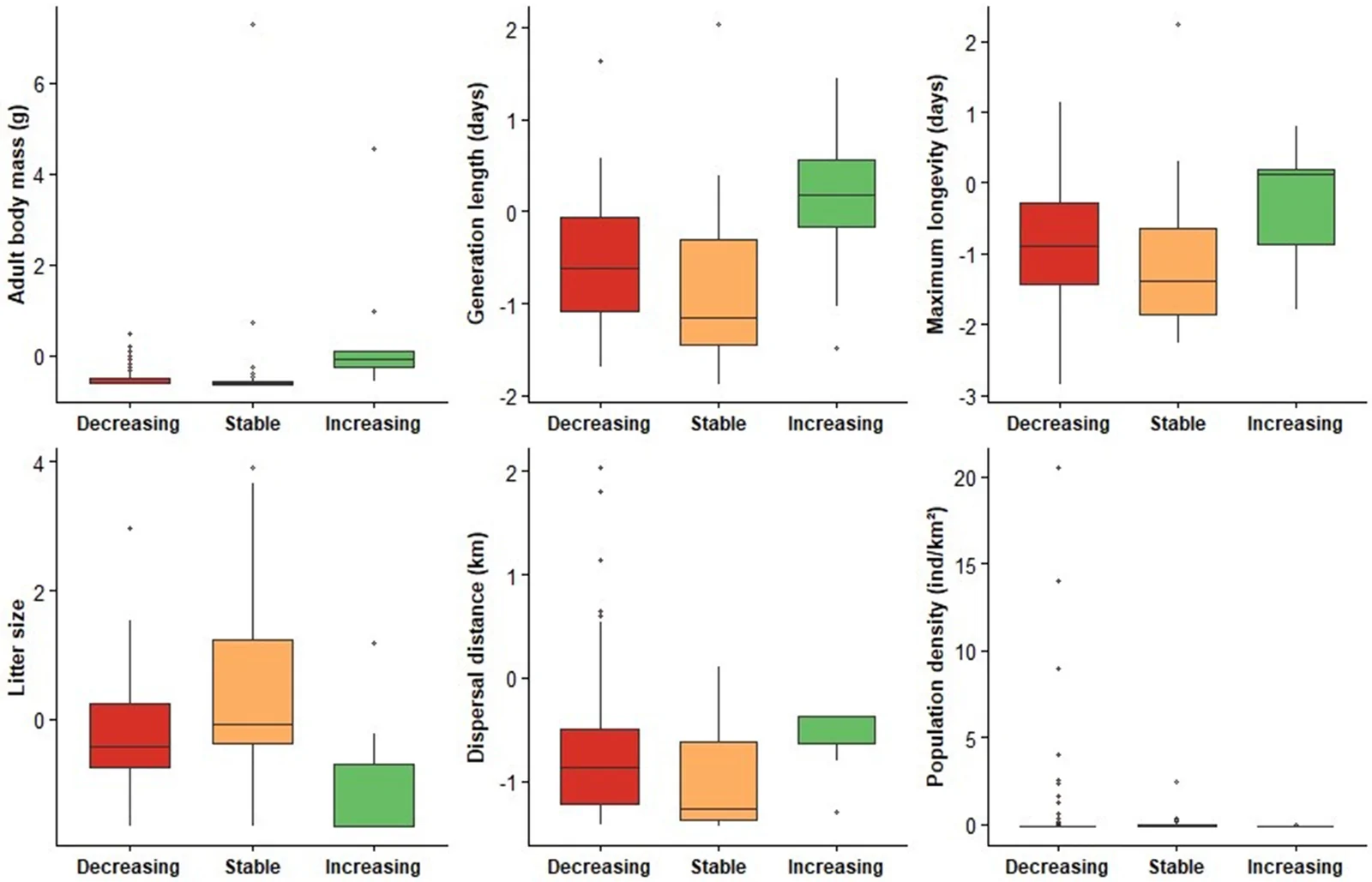
Variation in ecological and life-history traits across population trend categories. Boxplots illustrating variation in adult body mass, generation length, maximum longevity, litter size, dispersal distance, and population density among CITES-listed carnivores with decreasing, stable, and increasing population trends. Boxes represent the interquartile range (IQR), horizontal lines indicate medians, whiskers extend to 1.5 × IQR, and points represent outliers.

### Ecological traits associated with extinction risk

Logistic regression identified three ecological traits that were significantly associated with extinction risk (Table 2). Generation length was positively associated with threatened status (β = 0.940, OR = 2.56, p = 0.005), whereas dispersal distance showed the strongest positive association with threatened status (β = 1.660, OR = 5.26, p < 0.001). In contrast, habitat breadth was negatively associated with extinction risk (β = −0.927, OR = 0.40, p < 0.001). Litters per year and population density were retained in the final model but were not statistically significant (p > 0.05). The final model showed moderate discriminatory performance (AUC = 0.80), whereas additional model performance metrics and multicollinearity diagnostics are provided in the Supplementary Material (S1 and S2 Tables).

**Table 2:** Logistic regression model evaluating associations between ecological traits and threatened status in CITES-listed carnivores. Estimated regression coefficients (β), standard errors (SE), Wald z-statistics, p-values, odds ratios (OR), and 95% confidence intervals (95% CI) from the final binomial logistic regression model. Continuous predictor variables were standardized (z-scores) prior to analysis.

Predictor	β	SE	Z	p-value	OR	95% CI
Intercept	0.041	0.363	0.112	0.911	1.04	0.51–2.12
Generation length	0.940	0.337	2.793	**0.005**	2.56	1.32–4.95
Litters per year	0.441	0.242	1.823	0.068	1.55	0.97–2.50
Dispersal distance	1.660	0.498	3.335	**<0.001**	5.26	1.98–13.94
Population density	−0.263	0.198	−1.325	0.185	0.77	0.52–1.13
Habitat breadth	−0.927	0.251	−3.700	**<0.001**	0.40	0.24–0.65

### Family-level differences in extinction vulnerability

A bias-reduced logistic regression was used to evaluate differences in extinction risk among carnivore families (Fig 5). Using Canidae as the reference category, Ursidae exhibited significantly greater odds of threatened status (OR = 10.03, 95% CI = 1.41–71.57, p = 0.022). Although several additional families showed positive or negative estimated odds ratios relative to Canidae, their confidence intervals overlapped one and were not statistically significant. Complete regression coefficients are presented in S3 Table, and the number of species included in each family is provided in S4 Table to facilitate interpretation of family-level estimates.

**Fig 5:**
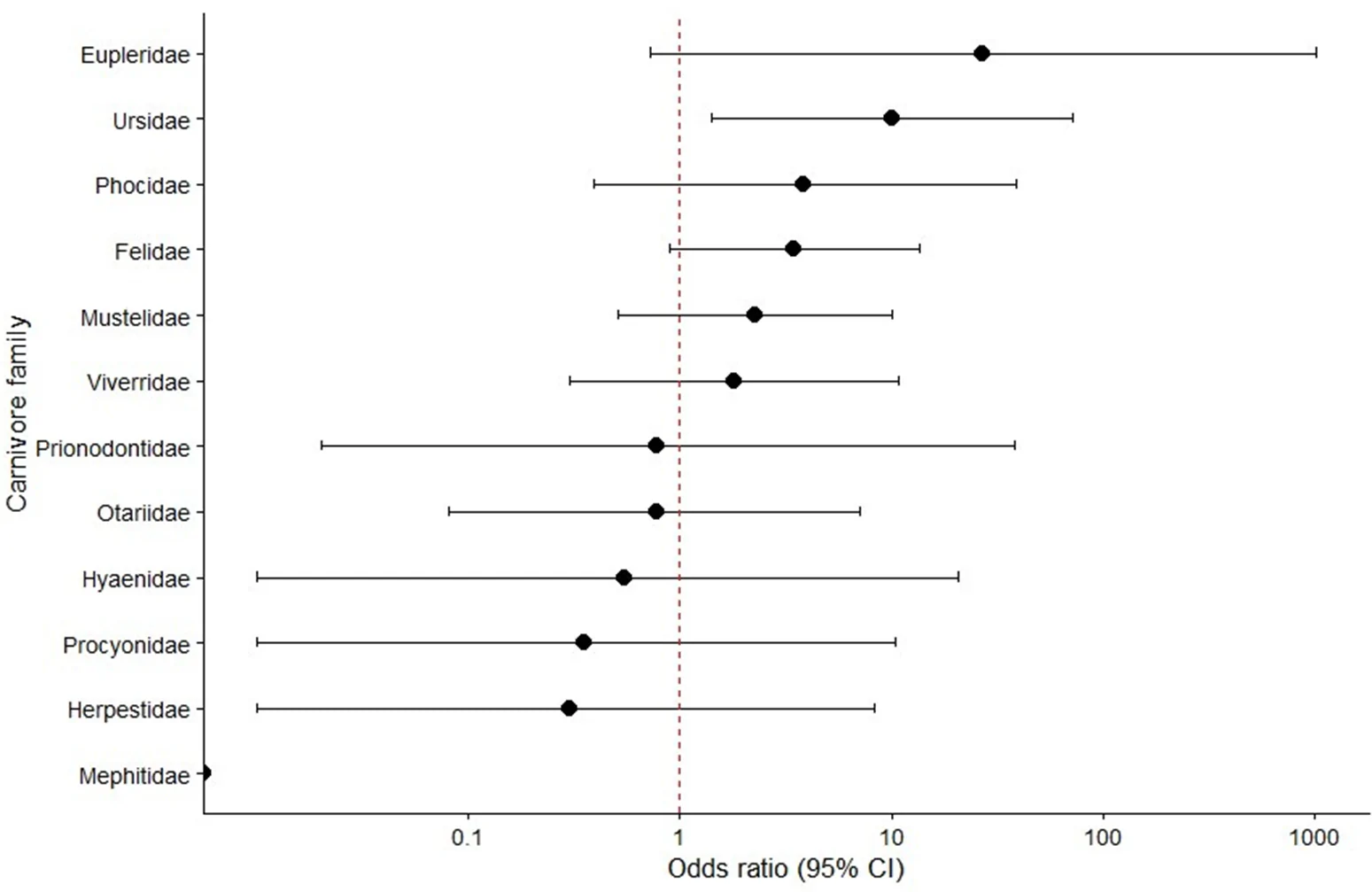
Family-level differences in extinction risk among CITES-listed carnivores. Odds ratios (OR) and 95% confidence intervals estimated from the bias-reduced logistic regression model. Odds ratios are shown relative to the reference family (Canidae). The vertical dashed line indicates OR = 1 (no difference from the reference family). Families with non-estimable infinite odds ratios resulting from complete separation were omitted from the figure for clarity but are reported in Table S3. The x-axis is presented on a logarithmic scale to improve visualization across the range of estimated odds ratios and confidence intervals.

## Discussion

Understanding why species differ in their extinction risk remains a central challenge in conservation biology [65,66]. By examining life-history traits within a globally standardized assemblage of CITES-listed carnivores, our study provides a macroecological framework for understanding patterns of extinction vulnerability among internationally traded species. Although our analyses did not quantify species-specific trade volumes or evaluate direct effects of trade on extinction risk, restricting the dataset to CITES-listed species allowed us to evaluate trait-based patterns within a globally standardized assemblage of internationally traded carnivores. Our results indicate that extinction risk among CITES-listed carnivores is associated with specific ecological traits. In particular, species with longer generation lengths and greater dispersal distances were more likely to be classified as threatened, whereas species occupying a broader range of habitats showed lower extinction risk. These findings are broadly consistent with previous macroecological studies showing that slow life histories and extensive spatial requirements increase extinction vulnerability [7,8,28]. Our results extend these well-established macroecological patterns to a globally representative assemblage of CITES-listed carnivores.

Our analyses of the spatial distribution of international trade records provide geographic context for the global distribution of reported trade involving CITES-listed carnivores. Several countries emerged as major importers and exporters throughout the study period. North American and European countries accounted for a large proportion of the reported international trade records, whereas Asian countries remained important import destinations throughout the study period. Previous studies have documented diverse forms of international wildlife trade involving carnivore species [67,68]. Documented examples include the international trade in lion (*Panthera leo*) bones [69] and the continued demand for Asian bears, including the Asian black bear (*Ursus thibetanus*), for bile and other body parts [42,70,71]. These examples provide broader context for the geographic patterns observed in our trade records but were not evaluated as mechanisms in the present analyses.

Our trait-based models identified ecological characteristics associated with threatened status among CITES-listed carnivores, providing a complementary basis for conservation assessment and monitoring. These trait associations could be used as an additional screening layer alongside information on trade intensity, specimen source, and population trends. For example, species with longer generation lengths may warrant closer evaluation when considered alongside reported trade volumes, specimen sources, and population trends, while recognizing that generation length may partly reflect body size [46,72]. Such a framework could help identify species warranting more detailed assessment or monitoring, while ecological traits should remain an additional line of evidence rather than a basis for assigning or changing IUCN Red List categories. Several studies have shown that market demand can increase as species become rarer under specific trade systems [73–75]. Our analyses focused largely on the legal, reported, international trade in carnivores, and we acknowledge that depending on the family there might be an additional illegal trade that we could not take into account. Likewise, we did not distinguish between different sources of traded specimens, such as wild-sourced, captive-bred, captive-born, or ranched individuals. These trade-related dimensions were not incorporated because the present analysis focused on species-level associations between ecological traits and IUCN extinction-risk status rather than on variation in trade composition or source. Future studies could benefit from integrating ecological traits with quantitative information on trade intensity, specimen source, and long-term population trajectories [13,76,77].

Our family-level analysis revealed limited differences in extinction risk among carnivore families. Relative to Canidae, only Ursidae exhibited significantly greater odds of threatened status. Although several additional families showed higher or lower estimated odds ratios, none differed significantly from the reference group. The wide confidence intervals observed for some families likely reflect their small sample sizes rather than strong biological differences. Differences among families may reflect well-established variation in life-history strategies, including body size, reproductive output, longevity, population density, and demographic resilience [5,7,8,28,38,71]. Collectively, these findings suggest that extinction vulnerability is primarily associated with species-specific ecological traits, while differences among families were comparatively limited.

Despite the robustness of our dataset and analytical framework, several limitations warrant consideration. CITES data exclude undocumented cross-border trade, illegal trade, and domestic trade; the latter is inherently difficult to quantify, and underreporting likely biases estimates of exploitation pressure for certain taxa and regions [78]. Moreover, our analyses were restricted to CITES-listed carnivores, which represent a subset of species regulated under international trade rather than the entire order Carnivora. Consequently, our findings should be interpreted within this taxonomic and regulatory scope and should not be generalized to non-CITES-listed carnivores. Additionally, variation in trait data availability across taxa may constrain analytical resolution. Some carnivore families were represented by relatively few species, resulting in wide confidence intervals and limited statistical power in the family-level analysis. Future research should prioritize longitudinal monitoring to detect temporal shifts in trade intensity and extinction risk, alongside socioeconomic investigations into evolving consumer demand. The development of integrated, real-time trade databases encompassing both legal and illegal flows would substantially improve our understanding of relationships between wildlife trade and extinction vulnerability.

Our findings reinforce the value of ecological traits as correlates of extinction vulnerability within CITES-listed carnivores and suggest that trait-based approaches can complement existing conservation assessments [4,7,8]. The population-trend patterns shown in Fig 4 further indicate that associations between individual traits and extinction-risk status should not be interpreted as direct predictors of population trajectories, because trait distributions overlap across decreasing, stable, and increasing population trends. Although CITES listings and IUCN assessments serve different conservation purposes, and their relationship was not evaluated in this study, integrating ecological traits with future analyses of trade intensity and long-term population trajectories could provide a more comprehensive basis for identifying species requiring closer monitoring.

In conclusion, our results show that well-established life-history correlates of extinction vulnerability are associated with threatened status within CITES-listed carnivores, demonstrating that ecological vulnerability is detectable within a globally traded assemblage. Even if these findings do not demonstrate that international wildlife trade causes or increases extinction risk, they identify ecological correlates of vulnerability within an assemblage defined by international trade. Distinguishing intrinsic ecological vulnerability from trade-driven effects can provide a stronger basis for integrating ecological traits with trade intensity, specimen source, and population trends in conservation assessment and monitoring.

## Supporting information

S1 Table Variance inflation factors for candidate ecological predictors. Variance inflation factors (VIFs) calculated for all candidate ecological predictors prior to model selection. All VIFs values were below the commonly used threshold of 5, indicating low multicollinearity among predictor variables.
(DOCX)

S2 Table Predictive performance of the final logistic regression model. Performance metrics of the final logistic regression model predicting extinction risk in CITES-listed carnivores.
(DOCX)

S3 Table Family-level differences in extinction risk estimated using bias-reduced logistic regression. Estimated regression coefficients (β), standard errors (SE), Wald z-statistics, p-values, odds ratios (OR), and 95% confidence intervals (95% CI) from the bias-reduced logistic regression model. Canidae was specified as the reference category. Infinite odds ratios resulted from complete separation caused by families represented by very few species and should be interpreted with caution.
(DOCX)

S4 Table Number of CITES-listed carnivore species included in each taxonomic family used in the family-level logistic regression analysis. Species are classified according to IUCN Red List status as threatened (Critically Endangered, Endangered, and Vulnerable) or non-threatened (Near Threatened and Least Concern).
(DOCX)
