# Supplementary material for: Ecological traits predict extinction risk of the world’s traded carnivores
Source: PLoS One. 2026 Nov 5;21(11):e0362196. doi: 10.1371/journal.pone.0362196 (PMC13739137; doi:10.1371/journal.pone.0362196)
Supplement: Supplementary file 1 [file pone.0362196.s001.docx]

**Supporting Information**

**Additional Supporting Information may be found in the online version of this article**.

**Table S1. Variance inflation factors for candidate ecological predictors.** Variance inflation factors (VIFs) calculated for all candidate ecological predictors prior to model selection. All VIFs values were below the commonly used threshold of 5, indicating low multicollinearity among predictor variables.

| **Predictor** | **VIF** |
| --- | --- |
| Adult body mass | 1.30 |
| Generation length | 3.59 |
| Maximum longevity | 2.87 |
| Litter size | 1.36 |
| Litters per year | 1.84 |
| Dispersal distance | 1.73 |
| Population density | 1.08 |
| Habitat breadth | 1.52 |
