# Supplementary material for: Ecological traits predict extinction risk of the world’s traded carnivores
Source: PLoS One. 2026 Nov 5;21(11):e0362196. doi: 10.1371/journal.pone.0362196 (PMC13739137; doi:10.1371/journal.pone.0362196)
Supplement: Supplementary file 2 [file pone.0362196.s002.docx]

**Table S2**. **Predictive performance of the final logistic regression model**. Performance metrics of the final logistic regression model predicting extinction risk in CITES-listed carnivores.

| **Metric** | **Value** |
| --- | --- |
| Area under the ROC curve (AUC) | 0.80 |
| Classification accuracy | 74.2% |
| Sensitivity | 54.3% |
| Specificity | 85.4% |
| Cohen's Kappa | 0.415 |
