# Supplementary material for: Ecological traits predict extinction risk of the world’s traded carnivores
Source: PLoS One. 2026 Nov 5;21(11):e0362196. doi: 10.1371/journal.pone.0362196 (PMC13739137; doi:10.1371/journal.pone.0362196)
Supplement: Supplementary file 3 [file pone.0362196.s003.docx]

**Table S3. Family-level differences in extinction risk estimated using bias-reduced logistic regression**. Estimated regression coefficients (β), standard errors (SE), Wald z-statistics, p-values, odds ratios (OR), and 95% confidence intervals (95% CI) from the bias-reduced logistic regression model. Canidae was specified as the reference category. Infinite odds ratios resulted from complete separation caused by families represented by very few species and should be interpreted with caution.

| **Family** | **β** | **SE** | **z** | **p-value** | **OR** | **95% CI** |
| --- | --- | --- | --- | --- | --- | --- |
| Intercept | -1.350 | 0.618 | -2.183 | 0.029 | 0.26 | 0.08–0.87 |
| Ailuridae | NA | NA | NA | NA | Infinite | Not estimable |
| Eupleridae | 3.296 | 1.852 | 1.780 | 0.075 | 27.00 | 0.72–1018.16 |
| Felidae | 1.247 | 0.698 | 1.786 | 0.074 | 3.48 | 0.89–13.68 |
| Herpestidae | -1.215 | 1.701 | -0.714 | 0.475 | 0.30 | 0.01–8.33 |
| Hyaenidae | -0.596 | 1.852 | -0.322 | 0.748 | 0.55 | 0.01–20.78 |
| Mephitidae | NA | NA | NA | NA | 0.00 | Not estimable |
| Mustelidae | 0.816 | 0.760 | 1.074 | 0.283 | 2.26 | 0.51–10.03 |
| Odobenidae | NA | NA | NA | NA | Infinite | Not estimable |
| Otariidae | -0.260 | 1.132 | -0.229 | 0.819 | 0.77 | 0.08–7.10 |
| Phocidae | 1.350 | 1.176 | 1.148 | 0.251 | 3.86 | 0.39–38.64 |
| Prionodontidae | -0.260 | 1.996 | -0.130 | 0.897 | 0.77 | 0.02–38.54 |
| Procyonidae | -1.048 | 1.732 | -0.605 | 0.545 | 0.35 | 0.01–10.46 |
| Ursidae | **2.305** | **1.003** | **2.299** | **0.022** | **10.03** | **1.41–71.57** |
| Viverridae | 0.588 | 0.918 | 0.640 | 0.522 | 1.80 | 0.30–10.89 |
