# Supplementary material for: Ecological traits predict extinction risk of the world’s traded carnivores
Source: PLoS One. 2026 Nov 5;21(11):e0362196. doi: 10.1371/journal.pone.0362196 (PMC13739137; doi:10.1371/journal.pone.0362196)
Supplement: Supplementary file 4 [file pone.0362196.s004.docx]

**Table S4. Number of CITES-listed carnivore species included in each taxonomic family used in the family-level logistic regression analysis.** Species are classified according to IUCN Red List status as threatened (Critically Endangered, Endangered, and Vulnerable) or non-threatened (Near Threatened and Least Concern).

| **Family** | **Total species** | **Threatened** | **Non-threatened** |
| --- | --- | --- | --- |
| Felidae | 38 | 18 | 20 |
| Mustelidae | 22 | 8 | 14 |
| Canidae | 16 | 3 | 13 |
| Viverridae | 10 | 3 | 7 |
| Otariidae | 8 | 1 | 7 |
| Ursidae | 8 | 6 | 2 |
| Herpestidae | 6 | 0 | 6 |
| Procyonidae | 5 | 0 | 5 |
| Phocidae | 4 | 2 | 2 |
| Eupleridae | 3 | 3 | 0 |
| Hyaenidae | 3 | 0 | 3 |
| Prionodontidae | 2 | 0 | 2 |
| Ailuridae | 1 | 1 | 0 |
| Mephitidae | 1 | 0 | 1 |
| Odobenidae | 1 | 1 | 0 |
